# Optimal and total controllability approach of non-instantaneous Hilfer fractional derivative with integral boundary condition

**DOI:** 10.1371/journal.pone.0297478

**Published:** 2024-02-28

**Authors:** Kottakkaran Sooppy Nisar, K. Jothimani, C. Ravichandran

**Affiliations:** 1 Department of Mathematics, College of Science and Humanities in Alkharj, Prince Sattam bin Abdulaziz University, Alkharj, Saudi Arabia; 2 Department of Mathematics, School of Advanced Sciences, Vellore Institute of Technology, Vellore, Tamil Nadu, India; 3 Department of Mathematics, Kongunadu Arts and Science College, Coimbatore, Tamil Nadu, India; University of Porto Faculty of Engineering: Universidade do Porto Faculdade de Engenharia, PORTUGAL

## Abstract

The focus of this work is on the absolute controllability of Hilfer impulsive non-instantaneous neutral derivative (HINND) with integral boundary condition of any order. Total controllability refers to the system’s ability to be controlled during the impulse time. Kuratowski measure and semigroup theory in Banach space yield the results. Furthermore, we talked about optimal controllability in conjunction with appropriate limitations. Our established outcomes are described using an example.

## 1 Introduction

The concept of differential equations with non-instantaneous impulses(NII) involves many physical processes due to its tremendous applications. Impulse is an action, that starts at an arbitrary fixed point and remains active on a finite time interval is called as NI impulse that occurs in many physical processes like harvesting, vaccination, natural disasters, and shocks subjected to unexpected change in their state. The above situations have to be modeled by impulses [1, 2] if necessary that can not be solved using ordinary differential equations. For some processes, instantaneous impulsive dynamic systems do not support a perfect description, for example, endorsement of insulin of hyperglycemia patients. The change in the above system caused by this medication will remain until the total absorption for a finite time, thanks to the evolutionary process can be modeled with NII. This theory is originated by Hernández [3]. Recently, Vipin Kumar et al. [4–7] derived the controllability results of fractional systems with and without NII for various models. To seek more about NI impulse, track and surf the articles [8–16] and cited references.

On the other hand, the existence and controllability theory extended for both DEs of integer and non-integer order with NII. Fractional calculus is the most appropriate way to evaluate the exact solutions to the given model. The results on Caputo and R-L fractional derivatives were discussed in [17–19]. Theory on HFD was introduced by Hilfer [20] and the results are discussed in [21–24]. One can refer to the monographs [25–29] to know more about fractional derivatives. In general, controllability enables directing the system from a random initial state to the desired ultimate state. The articles [30–35] discuss the controllability results of Caputo and Hilfer fractional differential system in the nondense domain. Furthermore, the existence and controllability of the Hilfer fractional system with infinite delay were examined in [36, 37]. The exact controllability for Hilfer fractional differential inclusions including nonlocal initial conditions was examined by Du et al. [38]. The approximate controllability results for the Hilfer fractional system were derived by [39, 40]. Recently, a prospective field in control systems is optimal control studied in [41–43]. Ultimately, is more appropriate to evaluate them using an optimization procedure involving fractional differential equations.

The outcome of the existence of HINND of arbitrary order was discussed in [44]. Moreover, results on total controllability fractional neutral non-instantaneous system discussed by [45]. In addition, optimization of the non-instantaneous neutral fractional system is investigated by in [46]. No article was found in the existing literature about the investigation of total controllability using semigroup theory.

We contribute this article to analyze the total controllability & optimal control results for HINND of arbitrary order as:

$$\begin{array}{ccc} & D_{e_{k}}^{p_{1},p_{2}}[z(t)-K(t,z(t))]=A[z(t)-K(t,z(t))]+Bu\left(t\right)+F(t,z(t)),\;t\in \overset{N}{\underset{k=0}{⋃}}\left(e_{k},t_{k+1}\right] & \\ & \;\;\;\;\;z\left(t\right)=\frac{1}{\Gamma (p_{1})}\int_{t_{k}}^{t}{\left(t-\omega \right)}^{p_{1}-1}I_{k}\left(\omega ,z\left(t_{k}^{-}\right)\right)d\omega ,\;t\in \overset{N}{\underset{k=1}{⋃}}\left(t_{k},e_{k}\right) & \\ & \;\;\;\;\;\;I_{0^{+}}^{\left(1-\eta \right)}z\left(0\right)=\lambda \int_{0}^{T}z\left(\omega \right)d\omega +c,\;c\in R. \end{array}$$ (1.1)

Here, Y be a Banach space and A:D(A)⊂Y→Y is closed together with D(A)∈Y. $D_{e_{k}}^{p_{1},p_{2}}$ represents Hilfer derivative of fractional order with 0 < *p*_1_ < 1, 0 ≤ *p*_2_ ≤ 1. Also, *η* = *p*_1_ + *p*_2_ − *p*_1_*p*_2_, t∈I=[0,T],T>0. Here K:I×Y→D(A)⊂Y, F:I×Y→D(A)⊂Y, $I_{k}:\left[t_{k},e_{k}\right]\times Y\to Y$ are relevant functions. $t_{k},e_{k}$ fulfills $0=t_{0}=e_{0}<t_{1}<e_{1}<t_{2}<\ldots .<e_{N}<t_{N+1}=T$. Moreover, $z\left(t_{k}^{-}\right)=\underset{h\to 0^{+}}{\text{lim}}z\left(t_{k}-h\right)$. B:U→Y is a bounded linear operator and $u\left(\cdot \right)\in L^{2}\left[I,U\right]$. The integral boundary condition λ = + 1 or −1. We briefly orchestrated our objective of this work:

(*i*) By incorporating HFD with semigroup operator theory and LT, we have introduced the integral solution of (1.1).
(*ii*) Kuratowski’s measure with *κ*–set-contraction theory has been supported very much to the total controllability of HINND with *C*_0_ semigroup operator for the first time in the literature.
(*iii*) The results on optimal controllability of HINND had been discussed via Lipschitz continuity.
(*iv*) We have gone through with an illustration that enables our analytical outcomes existence.

## 2 Key notes

The space of continuous functions is defined by C(I,Y) be a provided $\left||z|\right|=\underset{t\in I}{\text{sup}}||z\left(t\right)||.$

$C_{1-\eta }\left(I,Y\right)=\left\{z:Y\to Y\;\;\;\text{provided}\;\;\;t^{1-\eta }z\left(t\right)\in C\left(I,Y\right)\right\}$ , ${\left||z|\right|}_{C_{1-\eta }}=\underset{0\leq t\leq T}{\text{sup}}\left|t^{1-\eta }z\left(t\right)\right|$.

Let $PC_{1-\eta }\left(\left(t_{k},t_{k+1}\right],Y\right)$ defines the space of piecewise functions as

$$PC_{1-\eta }\left(I,Y\right)=\{\begin{array}{c} {\left(t-t_{k}\right)}^{1-\eta }z\left(t\right)\in C_{1-\eta }\left(\left(t_{k},t_{k+1}\right],R\right)\\ \underset{t\to t_{k}}{\text{lim}}{\left(t-t_{k}\right)}^{1-\eta }z\left(t\right),\;\;k=1,2,\ldots ,N, \end{array}\}$$

provided

$$\left||z|\right|=\text{max}\{\underset{t\in I}{\text{sup}}\left|\right|t^{1-\eta }z\left(t^{+}\right)||,\;\;\underset{t\in I}{\text{sup}}\left|\right|t^{1-\eta }z\left(t^{-}\right)\left|\right|\}.$$

L(Y), characterize the space of all bounded linear operators on Y. *A*, generates the semigroup $\left\{S_{p_{1},p_{2}}\left(t\right)\right\}$ where t≥0 with $\text{sup}||S_{p_{1},p_{2}}\left(t\right){\left|\right|}_{L(Y)}=M$. Define a convex, bounded and closed set $\ell =\{z\in PC_{1-\eta }\left(I,Y\right),\;\;||z\left(t\right)||<r,\;\;t\in I,\;\;r>0\}$ in $PC_{1-\eta }\left(I,Y\right)$.

**Definition 2.1** [20]. *For n* − 1 < *p*_1_ < *n*, *n* ∈ *N and p*_2_ ∈ (0, 1], *HFD is defined by*:

$$\begin{array}{ccc} & D_{0^{+}}^{p_{1},p_{2}}y\left(t\right)=I_{0^{+}}^{p_{1}\left(n-p_{2}\right)}\frac{d}{dt}I_{0^{+}}^{\left(1-p_{1}\right)\left(n-p_{2}\right)}y\left(t\right)=I_{0^{+}}^{p_{1}\left(n-p_{2}\right)}D_{0^{+}}^{p_{2}+p_{1}n-p_{2}p_{1}}y\left(t\right), & \end{array}$$

*where* $D_{0^{+}}^{p_{2}+p_{1}n-p_{2}p_{1}}$ *and* $I_{0^{+}}^{p_{1}\left(n-p_{2}\right)}$ *are R-L derivative and integral respectively*.

**Definition 2.2** [8, 44, 47]. *The Kuratowski noncompact measure ℓ*(⋅) *characterized as*: $\ell \left(\hbar \right)≔\text{inf}\{\rho >0:\hbar =\overset{m}{\underset{i=1}{⋃}}\;with\;diam\left(\hbar_{i}\right)\leq \rho \}$, *where ℏ is a bounded set on* Y.

**Lemma 2.3**. (see [8, 44, 47]) *For* $\hbar_{1},\hbar_{2}\subset Y$, *the Kuratowski noncompact measure meets*:

1.
$\ell \left(\hbar \right)=\ell \left(\bar{\hbar }\right)=\ell \left(conv\hbar \right);$

2. *ℓ*(ℏ) = 0 *iff* $\bar{\hbar }$ *is compact*;
3. *for given* λ ∈ *R*, *ℓ*(λℏ) ≤ |λ|*ℓ*(ℏ);
4. ℏ_1_ ⊂ ℏ_2_ *implies* *ℓ*(ℏ_1_) ≤ *ℓ*(ℏ_2_);
5. *ℓ*(ℏ_1_∪ℏ_2_) = max{*ℓ*(ℏ_1_), *ℓ*(ℏ_2_)};
6. *ℓ*(ℏ_1_ + ℏ_2_) ≤ *ℓ*(ℏ_1_) + *ℓ*(ℏ_2_), *where* $\hbar_{1}+\hbar_{2}=\left\{z\;|\;z=z_{1}+z_{2};\;\;z_{1}\in \hbar_{1},\;\;z_{2}\in \hbar_{2}\right\}$;
7. *The Lipschitz function* ℜ:D(ℜ)⊂Y→Y *and the subset* W⊂D(ℜ) , *ℓ*(ℜ(*W*)) ≤ *κ* *ℓ*(*W*) *is bounded*.

Let $D\subset C_{1-\eta }\left(I,Y\right)$ and t∈I, D(t)={z(t)|z∈D} and $\ell \left(D\left(t\right)\right)\leq \ell C_{1-\eta }\left(D\right)$.

**Lemma 2.4**. (*see* [8, 44, 47]) *Let* $D\subset C_{1-\eta }\left(\left[c_{1},c_{2}\right],Y\right)$ *be bounded and equicontinuous such that*

$$\ell C_{1-\eta }\left(D\right)=\underset{t\in [c_{1},c_{2}]}{\text{max}}\ell \left(D\left(t\right)\right),$$

*and* ℓ(D(t)) *is continuous on* [*c*_1_, *c*_2_].

**Lemma 2.5**. (*see* [8, 44, 47]) *Assume that* $\bar{Y}\subset Y$ *is bounded and for some* $D_{0}\subset D$, *the countable set meets* $\ell \left(D\right)\leq 2\;\ell \left(D_{0}\right)$.

**Lemma 2.6**. (*see* [8, 44, 47]) *Let* $D=\left\{z_{n}\right\}\subset PC_{1-\eta }\left(\left[c_{1},c_{2}\right],Y\right)$ *where* −∞ < *c*_1_ < *c*_2_ < ∞. *Hence* ℓ(D(t)) *on* [*c*_1_, *c*_2_] *such that*:

$$\ell (\{\int_{c_{1}}^{c_{2}}z_{n}\left(t\right)dt\})\leq 2\int_{c_{1}}^{c_{2}}\ell \left(D\left(t\right)\right)dt,\;\;\;n\in N.$$

**Lemma 2.7**. (*see* [21, 22, 44]) *The system* (1.1) *becomes*:

$$\begin{array}{cc} z(t) & =\frac{t^{\eta -1}}{\Gamma (\eta )}[\lambda \int_{0}^{T}z\left(\omega \right)d\omega +c-K\left(0,z\left(0\right)\right)]+K\left(t,z\left(t\right)\right)\\ & +\frac{1}{\Gamma (p_{1})}\int_{0}^{t}{\left(t-\omega \right)}^{p_{1}-1}[A[z(\omega )-K(\omega ,z(\omega ))]+Bu\left(\omega \right)+F\left(\omega ,z\left(\omega \right)\right)]d\omega ,\;t\in \left(0,t_{1}\right],\\ z(t) & =\frac{1}{\Gamma (p_{1})}\int_{t_{k}}^{t}{\left(t-\omega \right)}^{p_{1}-1}I_{k}\left(\omega ,z\left(t_{k}^{-}\right)\right)d\omega ,\;t\in \left(t_{k},e_{k}\right],\\ z(t) & =\frac{1}{\Gamma (p_{1})}\int_{t_{k}}^{e_{k}}{\left(t-\omega \right)}^{p_{1}-1}I_{k}\left(\omega ,z\left(t_{k}^{-}\right)\right)d\omega -K\left(e_{k},z\left(e_{k}\right)\right)+K\left(t,z\left(t\right)\right)\\ & +\frac{1}{\Gamma (p_{1})}\int_{e_{k}}^{t}{\left(t-\omega \right)}^{p_{1}-1}[A[z(\omega )-K(\omega ,z(\omega ))]\\ & +Bu\left(\omega \right)+F\left(\omega ,z\left(\omega \right)\right)]d\omega ,\;t\in \left(e_{k},t_{k+1}\right]. \end{array}$$

**Definition 2.8**. (*see* [21, 22, 44]) *A function* $z\in PC_{1-\eta }\left(I,Y\right)$ *is a solution of* (1.1), *if*

(*i*) $z\left(t\right)=\frac{1}{\Gamma (p_{1})}\int_{t_{k}}^{t}{\left(t-\omega \right)}^{p_{1}-1}I_{k}\left(\omega ,z\left(t_{k}^{-}\right)\right)d\omega ,\;\;\;t\in \left(t_{k},e_{k}\right],\;\;k=1,2,...,N$

(*ii*) $I_{0}^{1-\eta }z\left(0\right)=\lambda \int_{0}^{T}z\left(\omega \right)d\omega +c$ ,

*together with*

$$\begin{array}{ccc} z(t) & =S_{p_{1},p_{2}}\left(t\right)[\lambda \int_{0}^{T}z\left(\omega \right)d\omega +c-K\left(0,z\left(0\right)\right)]+K\left(t,z\left(t\right)\right)\\ & +\int_{0}^{t}K_{p_{1}}\left(t-\omega \right)[Bu(\omega )+F(\omega ,z(\omega ))]d\omega ,\;t\in \left(0,t_{1}\right], & \end{array}$$ (2.1)

$$\begin{array}{cc} z(t) & =S_{p_{1},p_{2}}\left(t-e_{k}\right)[\frac{1}{\Gamma (p_{1})}\int_{t_{k}}^{e_{k}}{\left(e_{k}-\omega \right)}^{p_{1}-1}I_{k}\left(\omega ,z\left(t_{k}^{-}\right)\right)d\omega -K\left(e_{k},z\left(e_{k}\right)\right)]+K\left(t,z\left(t\right)\right)\\ & +\int_{e_{k}}^{t}K_{p_{1}}\left(t-\omega \right)[Bu(\omega )+F(\omega ,z(\omega ))]d\omega ,\;t\in \left(e_{k},t_{k+1}\right],\;k=1,2,...,N. \end{array}$$ (2.2)

$$T_{p_{1}}\left(t\right)=p_{1}\int_{0}^{\infty }\nu \psi_{y}\left(\nu \right)ℜ\left(t^{p_{1}}\nu \right)d\nu ,\;K_{p_{1}}\left(t\right)=t^{p_{1}-1}T_{p_{1}}\left(t\right),\;S_{p_{1},p_{2}}\left(t\right)=I_{0^{+}}^{p_{2}\left(1-p_{1}\right)}K_{p_{1}}\left(t\right).$$

$$\begin{array}{ccc} W_{y}\left(\nu \right) & = & \frac{1}{\pi }\sum_{m=1}^{\infty }{\left(-1\right)}^{m-1}\nu^{-my-1}\frac{\Gamma (my+1)}{m!}\text{sin}\left(m\pi z\right),\;\;\;\nu \in \left(0,\infty \right), \end{array}$$

$$\begin{array}{ccc} \psi_{y}\left(\nu \right) & = & \frac{1}{z}\nu^{\left(-1-\frac{1}{z}\right)}W_{y}\left(\nu^{-\frac{1}{z}}\right)\geq 0. \end{array}$$

**Lemma 2.9**. (*see* [8, 44, 47]) *If a family* $\{S_{p_{1}}\left(t\right),\;t\geq 0\}\subset B\left(Y\right)$ *satisfies*

(*i*) *for all* z∈D(A) , $S_{p_{1}}\left(t\right)z=z+I_{0^{+}}^{p_{1}}\;\;S_{p_{1}}\left(t\right)Ay$, t≥0;
(*ii*) $S_{p_{1}}\left(t\right)$ *is strongly continuous on* $R_{+}$ , $S_{p_{1}}\left(0\right)=I;$
(*iii*) $AS_{p_{1}}\left(t\right)z=S_{p_{1}}A\left(z\right)$ *for each*, z∈D(A),t≥0, D⊂Y.

*Then, it is said to be* *p*_1_–*times resolvent generator by A*.

**Definition 2.10**. *A system is defined as totally controllable on* I, *if for k* = 1, 2, …, *N*, *it is controllable on* $\left(0,t_{1}\right]$, $\left(e_{k},t_{k+1}\right]$ such that $z\left(0\right)=z_{0}$ and $z\left(t_{k+1}\right)=z_{t_{k+1}}$.

For further discussions, we consider the subsequent assumptions as:

(*H*1) $K:J_{0}\times Y\to Y,\;\;J_{0}=\overset{N}{\underset{k=0}{⋃}}\left(e_{k},t_{k+1}\right]$ is continuous and for $L_{p},\mu_{p}>0$ as
  $$||K\left(t,\hat{z_{2}}\right)-K\left(t,\hat{z_{1}}\right)\left|\right|\leq L_{p}\left|\right|\hat{z_{2}}-\hat{z_{1}}\left|\right|,\;\;\;\hat{z_{2}},\;\;\hat{z_{1}}\in Y,\;\;\;t\in J_{0},$$
  also $||K(t,z||\leq \mu_{p},\;\;z\in Y,\;\;t\in J_{0};$
(*H*2) for any bounded set $D_{1}\subset Y$, *exist* $L_{p^{*}}>0$, such that
  $$\ell \left(K\left(t,D_{1}\right)\right)\leq L_{p^{*}}\ell \left(D_{1}\right);$$
(*H*3) Function $F:J_{0}\times Y\to Y$ is continuous with $L_{f}>0$, satisfies
  $$||F\left(t,\hat{z_{1}}\right)-F\left(t,\hat{z_{2}}\right)\left|\right|\leq L_{f}\left|\right|\hat{z_{1}}-\hat{z_{2}}\left|\right|,\;\;\;\hat{z_{1}},\;\hat{z_{2}}\in Y,\;\;t\in J_{0}.$$
  $$\left||F\left(t,z\right)|\right|\leq \Psi \left(t\right)P(||z||)\;\;\;\text{and}\;\;\;\underset{l\to \infty }{\text{lim}}\text{inf}\frac{P(l)}{l}=\nu <\infty ;$$
  where ℘ : [0, ∞)→[0, ∞), a non decreasing continuous function, ψ:I→[0,∞), a Lebesgue integrable function and *ν* > 0 such that for all z∈Y, t∈I and meets ${\left||z|\right|}_{C_{1-\eta }}\leq l$.
(*H*4) For $k=0,1,\ldots N,L_{k}$,
  $$\ell \left(F\left(t,z\right)\right)\leq L_{k}\;\ell \left(D\right),\;\;\;t\in J_{0}\;\;\;\text{with}\;\;\;L=\underset{k}{\text{max}}\;L_{k},$$
  where the subset *D* of Y is a countable;
(*H*5) For $J_{k}=\left[t_{k},e_{k}\right],\;\;k=1,2,\ldots N$, $I_{k}:J_{k}\times Y\to Y$ are continuous functions, for $K_{I_{k}}>0,\;\;k=1,2,\ldots N$, provided for every $\hat{z_{1}},\;\hat{z_{2}}\in Y$,
  $$\left|\right|I_{k}\left(t,\hat{z_{1}}\right)-I_{k}\left(t,\hat{z_{2}}\right)\left|\right|\leq K_{I_{k}}\left|\right|\hat{z_{1}}-\hat{z_{2}}\left|\right|,\;\;\;\text{for}\;\text{each}\;\;\;t\in \left(t_{k},e_{k}\right],\;\;\;K≔\underset{k=0,1,\ldots N}{\text{max}}\;K_{I_{k}}.$$
  Moreover, $M_{I}$, together with $\left|\right|I_{k}\left(t,z\right)\left|\right|\leq M_{I};$
(*H*6) $W:L^{2}\left(I,U\right)\to Y$ defined by:
  $$Wu=\int_{0}^{a}K_{p_{1}}\left(a-\omega \right)Bu\left(\omega \right)d\omega ,$$
  is invertible. Also, for $M_{b},M_{w}\geq 0$, and $\|W^{-1}\|\leq M_{w}$, $\left\|B\right\|\leq M_{b}$.
(*H*7) Given ${L_{u}}^{*}>0$, for $\ell \left(u\left(z,\mu \right)\right)\leq {L_{u}}^{*}t^{1-\eta }\sigma \left(z,\mu \right)\ell \left(z\left(\mu \right)\right)$, a.e. μ∈I and $\underset{t\in I}{\text{sup}}\int_{0}^{t}\sigma \left(t,\mu \right)d\omega =\sigma^{*}<\infty$.

Conveniently, we assign some notations as follows:

$$\begin{array}{ccc} & C_{2}=\frac{M_{1}M_{b}M_{w}^{m}T^{p_{1}}}{p_{1}};\;k_{1}=\text{max}\{M\lambda T+L_{p},\;\;M(\frac{K_{I_{k}}t_{k+1}^{p_{1}}}{\Gamma (p_{1}+1)}+L_{p})+L_{p},\;\;\frac{K_{I_{k}}t_{k+1}^{p_{1}}}{\Gamma (p_{1}+1)}\}; & \\ & N_{1}=M(\frac{M_{I}t_{k+1}^{p_{1}}}{\Gamma (p_{1}+1)}+\mu_{p})+\mu_{p}+\frac{M_{1}t_{k+1}^{p_{1}}}{p_{1}}{P\left(l\right)||\Psi ||}_{L[0,t_{k+1}]};\;C_{1}=\frac{M_{1}M_{b}M_{w}^{1}t_{1}^{p_{1}}}{p_{1}}; & \\ & M_{1}=\text{sup}||K_{p_{1}}{\left(t\right)||}_{L(Y)};\;N=M(\lambda ||z||+\left|c\right|+\mu_{p})+\mu_{p}+\frac{M_{1}{t_{1}}^{p_{1}}}{p_{1}}{P\left(l\right)||\Psi ||}_{L[0,t_{1}]}. \end{array}$$

## 3 Main sequels

**Lemma 3.1**. *Let* S⊂Y *and* ℜ:S→Y *be called as κ-set-contractive for any bounded set* ℵ *in* S *such that and for κ* ∈ [0, 1), *as*

ℓ(ℜ(ℵ))≤κℓ(ℵ).

**Lemma 3.2**. *Let* ℵ *be a convex, bounded and closed subset of* Y. *If* ℜ : ℵ → ℵ *is κ-set-contractive. Then* ℜ *has at least one fixed point in* ℵ.

**Lemma 3.3**. *If the assumptions* (*H*1)–(*H*7) *true, hence*

$$\begin{array}{cc} u(t) & =W^{-1}[z_{t_{1}}-S_{p_{1},p_{2}}\left(t_{1}\right)[\lambda \int_{0}^{T}z\left(\omega \right)d\omega +c-K\left(0,z\left(0\right)\right)]-K\left(t_{1},z\left(t_{1}\right)\right)\\ & -\int_{0}^{t_{1}}K_{p_{1}}\left(t_{1}-\omega \right)F\left(\omega ,z\left(\omega \right)\right)d\omega ],\;\;\;t\in \left(0,t_{1}\right], \end{array}$$ (3.1)

$$\begin{array}{cc} u(t) & =\;\;W^{-1}[z_{t_{k+1}}-S_{p_{1},p_{2}}\left(t_{k+1}-e_{k}\right)\\ & \left(\times \right)[\frac{1}{\Gamma (p_{1})}\int_{t_{k}}^{e_{k}}{\left(e_{k}-\omega \right)}^{p_{1}-1}I_{k}\left(\omega ,z\left(t_{k}^{-}\right)\right)d\omega -K\left(e_{k},z\left(e_{k}\right)\right)]-K\left(t_{k+1},z\left(t_{k+1}\right)\right)\\ & -\int_{e_{k}}^{t_{k+1}}K_{p_{1}}\left(t_{k+1}-\omega \right)F\left(\omega ,z\left(\omega \right)\right)d\omega ],\;\;\;t\in \left(e_{k},t_{k+1}\right], \end{array}$$ (3.2)

*drives to* z(t) *of* (1.1) *from* $z\left(t_{1}\right)=z_{t_{1}}$ *and* $z\left(t_{k+1}\right)=z_{t_{k+1}}$, *also* $\left||u\left(t\right)|\right|=M_{u}^{1}$, $\left||u\left(t\right)|\right|=M_{u}^{k}$ *with*

$$M_{u}^{1}=M_{w}^{1}(\left|\right|z_{t_{1}}\left|\right|+N),\;\;\;M_{u}^{k}=M_{w}^{m}(\left|\right|z_{t_{k+1}}\left|\right|+N_{1}),\;\;k=1,2,\ldots ,N.$$

*Proof*. For $t=t_{1}$,

$$\begin{array}{cc} z(t_{1}) & =S_{p_{1},p_{2}}\left(t_{1}\right)[\lambda \int_{0}^{T}z\left(\omega \right)d\omega +c-K\left(0,z\left(0\right)\right)]+K\left(t_{1},z\left(t_{1}\right)\right)\\ & +\int_{0}^{t_{1}}K_{p_{1}}\left(t_{1}-\omega \right)F\left(\omega ,z\left(\omega \right)\right)d\omega \\ & +\int_{0}^{t_{1}}K_{p_{1}}\left(t_{1}-\tau \right)W^{-1}[z_{t_{1}}-S_{p_{1},p_{2}}\left(t_{1}\right)[\lambda \int_{0}^{T}z\left(\omega \right)d\omega +c-K\left(0,z\left(0\right)\right)]\\ & -K\left(t_{1},z\left(t_{1}\right)\right)-\int_{0}^{t_{1}}K_{p_{1}}\left(t_{1}-\omega \right)F\left(\omega ,z\left(\omega \right)\right)d\omega ]d\tau \\ & =S_{p_{1},p_{2}}\left(t_{1}\right)[\lambda \int_{0}^{T}z\left(\omega \right)d\omega +c-K\left(0,z\left(0\right)\right)]+K\left(t_{1},z\left(t_{1}\right)\right)\\ & +\int_{0}^{t_{1}}K_{p_{1}}\left(t_{1}-\omega \right)F\left(\omega ,z\left(\omega \right)\right)d\omega +z_{t_{1}}-S_{p_{1},p_{2}}\left(t_{1}\right)[\lambda \int_{0}^{T}z\left(\omega \right)d\omega +c-K\left(0,z\left(0\right)\right)]\\ & -K\left(t_{1},z\left(t_{1}\right)\right)-\int_{0}^{t_{1}}K_{p_{1}}\left(t_{1}-\omega \right)F\left(\omega ,z\left(\omega \right)\right)d\omega \\ & =z_{t_{1}}, \end{array}$$

with

$$\begin{array}{cc} \left||u(t)|\right| & \leq ||W^{-1}[z_{t_{1}}-S_{p_{1},p_{2}}\left(t_{1}\right)[\lambda \int_{0}^{T}z\left(\omega \right)d\omega +c-K\left(0,z\left(0\right)\right)]-K\left(t_{1},z\left(t_{1}\right)\right)\\ & -\int_{0}^{t_{1}}K_{p_{1}}\left(t_{1}-\omega \right)F\left(\omega ,z\left(\omega \right)\right)d\omega ]||\\ & \leq M_{w}^{1}\left(|\right|z_{t_{1}}\left|\right|+M||\lambda \int_{0}^{T}z\left(\omega \right)d\omega +c-K\left(0,z\left(0\right)\right)||+||K\left(t_{1},z\left(t_{1}\right)\right)||\\ & +M_{1}||\int_{0}^{t_{1}}\omega^{1-\eta }\Psi \left(\omega \right)P({\left||z|\right|}_{C_{1-\eta }})d\omega ||)\\ & \leq M_{w}^{1}(\left|\right|z_{t_{1}}\left|\right|+M(\lambda T||z\left(\omega \right)||+\left|c\right|+\mu_{p})+\mu_{p}+\frac{M_{1}t_{1}^{p_{1}}}{p_{1}}{P\left(l\right)||\Psi ||}_{L[0,t_{1}]})\\ & \leq M_{w}^{1}(\left|\right|z_{t_{1}}\left|\right|+N)\\ & =M_{u}^{1}. \end{array}$$

Also, for $t\in (e_{k},t_{k+1}]$ and $t=t_{k+1}$,

$$\begin{array}{cc} z(t_{k+1}) & =S_{p_{1},p_{2}}\left(t_{k+1}-e_{k}\right)[\frac{1}{\Gamma (p_{1})}\int_{t_{k}}^{e_{k}}{\left(e_{k}-\omega \right)}^{p_{1}-1}I_{k}\left(\omega ,z\left(t_{k}^{-}\right)\right)d\omega -K\left(e_{k},z\left(e_{k}\right)\right)]\\ & +K\left(t_{k+1},z\left(t_{k+1}\right)\right)+\int_{e_{k}}^{t_{k+1}}K_{p_{1}}\left(t_{k+1}-\omega \right)F\left(\omega ,z\left(\omega \right)\right)d\omega \\ & +\int_{e_{k}}^{t_{k+1}}K_{p_{1}}\left(t_{k+1}-\tau \right)W^{-1}[z_{t_{k+1}}-S_{p_{1},p_{2}}\left(t_{k+1}-e_{k}\right)\\ & \left(\times \right)[\frac{1}{\Gamma (p_{1})}\int_{t_{k}}^{e_{k}}{\left(e_{k}-\omega \right)}^{p_{1}-1}I_{k}\left(\omega ,z\left(t_{k}^{-}\right)\right)d\omega -K\left(e_{k},z\left(e_{k}\right)\right)]\\ & -K\left(t_{k+1},z\left(t_{k+1}\right)\right)-\int_{e_{k}}^{t_{k+1}}K_{p_{1}}\left(t_{k+1}-\omega \right)F\left(\omega ,z\left(\omega \right)\right)d\omega ]d\tau \\ & =z_{t_{k+1}}, \end{array}$$

with

$$\begin{array}{cc} \left||u(t)|\right| & \leq M_{w}^{m}(\left|\right|z_{t_{k+1}}\left|\right|+M(\frac{M_{I}t_{k+1}^{p_{1}}}{\Gamma (p_{1}+1)}+\mu_{p})+\mu_{p}+\frac{M_{1}t_{k+1}^{p_{1}}}{p_{1}}{P\left(l\right)||\Psi ||}_{L[0,t_{k+1}]})\\ & \leq M_{w}^{m}(\left|\right|z_{t_{k+1}}\left|\right|+N_{1})\\ & =M_{u}^{m}. \end{array}$$

**Theorem 3.4**. *The system* (1.1) *is totally controllable on* I, *if it meets the assumptions* (*H*1)–(*H*7) *together with the conditions*

$$\begin{array}{cc} & [M\lambda +2L_{p}^{*}+M_{I}\left(M+1\right)+4L_{u}^{*}\sigma^{*}M_{1}M_{b}M_{w}+4M_{1}L_{k}]<1. \end{array}$$ (3.3)

*Proof*. Construct $G:PC_{1-\eta }\left(I,Y\right)\to PC_{1-\eta }\left(I,Y\right)$ as

$$\left(Gz\right)\left(t\right)=\{\begin{array}{c} S_{p_{1},p_{2}}\left(t\right)[\lambda \int_{0}^{T}z\left(\omega \right)d\omega +c-K\left(0,z\left(0\right)\right)]+K\left(t,z\left(t\right)\right)\\ +\int_{0}^{t}K_{p_{1}}\left(t-\omega \right)[Bu(\omega )+F(\omega ,z(\omega ))]d\omega ,\;t\in \left(0,t_{1}\right];\\ \frac{1}{\Gamma (p_{1})}\int_{t_{k}}^{t}{\left(t-\omega \right)}^{p_{1}-1}I_{k}\left(\omega ,z\left(t_{k}^{-}\right)\right)d\omega ,\;t\in \left(t_{k},e_{k}\right];\\ S_{p_{1},p_{2}}\left(t-e_{k}\right)[\frac{1}{\Gamma (p_{1})}\int_{t_{k}}^{e_{k}}{\left(e_{k}-\omega \right)}^{p_{1}-1}I_{k}\left(\omega ,z\left(t_{k}^{-}\right)\right)d\omega -K\left(e_{k},z\left(e_{k}\right)\right)]\\ +K\left(t,z\left(t\right)\right)+\int_{e_{k}}^{t}K_{p_{1}}\left(t-\omega \right)[Bu(\omega )+F(\omega ,z(\omega ))]d\omega ,\;t\in \left(e_{k},t_{k+1}\right], \end{array}$$

where u(t) is described in (3.1) and (3.2) for $\left(0,t_{1}\right]$ and $\left(e_{k},t_{k+1}\right]$, respectively. Moreover, by Lemma 3.1, $z\left(t_{1}\right)=z_{t_{1}}$ and $z\left(t_{k+1}\right)=z_{t_{k+1}},\;\;k=1,2,\ldots ,N$. Let ${ℵ}_{\gamma }=\{z\in PC_{1-\eta }{\left(I,Y\right):||z||}_{PC_{1-\eta }}\leq \gamma \}\subseteq PC_{1-\eta }\left(I,Y\right),\;\;\;\gamma >0$, and

$$\gamma >\text{max}\{N+C_{1}(\left|\right|z_{t_{1}}\left|\right|+N),\underset{k=1,2,\ldots ,N}{\text{max}}\left\{N_{1}+C_{2}(\left|\right|z_{t_{k+1}}\left|\right|+N_{1})\right\},\;\frac{M_{I}T^{p_{1}}}{\Gamma (p_{1}+1)}\}.$$

**Step 1:** $G:{ℵ}_{\gamma }\to {ℵ}_{\gamma }$ .

For $t\in (0,t_{1}]$, let $z\in {ℵ}_{\gamma }$

$$\begin{array}{cc} \left||(Gz)(t)|\right| & \leq \left||S_{p_{1},p_{2}}\left(t\right)[\lambda \int_{0}^{T}z\left(\omega \right)d\omega +c-K\left(0,z\left(0\right)\right)]||+||K\left(t,z\left(t\right)\right)|\right|\\ & +\left||\int_{0}^{t}K_{p_{1}}\left(t-\omega \right)F\left(\omega ,z\left(\omega \right)\right)d\omega ||+||\int_{0}^{t}K_{p_{1}}\left(t-\omega \right)Bu\left(\omega \right)d\omega |\right|\\ & \leq M(\lambda T||z||+\left|c\right|+\mu_{p})+\mu_{p}+\frac{M_{1}t_{1}^{p_{1}}}{p_{1}}{P\left(l\right)||\Psi ||}_{L[0,t_{1}]}+\frac{M_{1}M_{b}M_{u}^{1}t^{p_{1}}}{p_{1}}\\ & \leq N+\frac{M_{1}M_{b}M_{w}^{1}t^{p_{1}}}{p_{1}}[\left|\right|z_{t_{1}}\left|\right|+N]\\ & \leq \gamma . \end{array}$$ (3.4)

Also, for $t\in (e_{k},t_{k+1}]$,

$$\begin{array}{cc} \left||(Gz)(t)|\right| & \leq \left||S_{p_{1},p_{2}}\left(t-e_{k}\right)[\frac{1}{\Gamma (p_{1})}\int_{t_{k}}^{e_{k}}{\left(e_{k}-\omega \right)}^{p_{1}-1}I_{k}\left(\omega ,z\left(t_{k}^{-}\right)\right)d\omega -K\left(e_{k},z\left(e_{k}\right)\right)]|\right|\\ & +\left||K\left(t,z\left(t\right)\right)||+||\int_{e_{k}}^{t}K_{p_{1}}\left(t-\omega \right)F\left(\omega ,z\left(\omega \right)\right)d\omega ||+||\int_{e_{k}}^{t}K_{p_{1}}\left(t-\omega \right)Bu\left(\omega \right)d\omega |\right|\\ & \leq M(\frac{M_{I}t_{k+1}^{p_{1}}}{\Gamma (p_{1}+1)}+\mu_{p})+\mu_{p}+\frac{M_{1}t_{1}^{p_{1}}}{p_{1}}{P\left(l\right)||\Psi ||}_{L[0,t_{k+1}]}+\frac{M_{1}M_{b}M_{u}^{1}T^{p_{1}}}{p_{1}}\\ & \leq N_{1}+\frac{M_{1}M_{b}M_{w}^{m}T^{p_{1}}}{p_{1}}(\left|\right|z_{t_{k+1}}\left|\right|+N_{1})\\ & \leq \gamma . \end{array}$$ (3.5)

Also, for $t\in (t_{k},e_{k}]$, and $z\in {ℵ}_{\gamma }$,

$$\begin{array}{cc} & \left||\left(Gz\right)\left(t\right)|\right|\leq \frac{M_{I}t_{k+1}^{p_{1}}}{\Gamma (p_{1}+1)}\leq \gamma . \end{array}$$ (3.6)

Hence, from (3.4)–(3.6), for some t∈I, gives ${\left||\left(Gz\right)\left(t\right)|\right|}_{PC_{1-\eta }}\leq \gamma$. Then $G:{ℵ}_{\gamma }\to {ℵ}_{\gamma }$.

Construct $G_{1}$, $G_{2}$ as:

$$\left(G_{1}y\right)\left(t\right)=\{\begin{array}{c} S_{p_{1},p_{2}}\left(t\right)[\lambda \int_{0}^{T}z\left(\omega \right)d\omega +c-K\left(0,z\left(0\right)\right)]+K\left(t,z\left(t\right)\right),\;t\in \left(0,t_{1}\right],\\ \frac{1}{\Gamma (p_{1})}\int_{t_{k}}^{t}{\left(t-\omega \right)}^{p_{1}-1}I_{k}\left(\omega ,z\left(t_{k}^{-}\right)\right)d\omega ,\;t\in \left(t_{k},e_{k}\right],\;\;k=1,2,\ldots ,N,\\ S_{p_{1},p_{2}}\left(t-e_{k}\right)[\frac{1}{\Gamma (p_{1})}\int_{t_{k}}^{e_{k}}{\left(e_{k}-\omega \right)}^{p_{1}-1}I_{k}\left(\omega ,z\left(t_{k}^{-}\right)\right)d\omega -K\left(e_{k},z\left(e_{k}\right)\right)]\\ +K\left(t,z\left(t\right)\right),\;t\in \left(e_{k},t_{k+1}\right],\;\;k=1,2,\ldots ,N, \end{array}$$

and

$$\left(G_{2}y\right)\left(t\right)=\{\begin{array}{cc} \int_{0}^{t}K_{p_{1}}\left(t-\omega \right)[Bu(\omega )+F(\omega ,z(\omega ))]d\omega , & t\in (0,t_{1}],\\ 0, & t\in (t_{k},e_{k}],\;\;\\ \int_{e_{k}}^{t}K_{p_{1}}\left(t-\omega \right)[Bu(\omega )+F(\omega ,z(\omega ))]d\omega , & t\in (e_{k},t_{k+1}]. \end{array}$$

Clearly, $G=G_{1}+G_{2}$.

**Step 2:** $G_{1}$ is contraction.

Let $z\in {ℵ}_{\gamma }$, for any $t\in (0,t_{1}]$,

$$\begin{array}{cc} \left|\right|[\left(G_{1}z_{1}\right)\left(t\right)-\left(G_{1}z_{2}\right)\left(t\right)]\left|\right| & =\left||S_{p_{1},p_{2}}\left(t\right)(\lambda \int_{0}^{T}z_{1}\left(\omega \right)d\omega -\lambda \int_{0}^{T}z_{2}\left(\omega \right)d\omega )|\right|\\ & +||K\left(t,z_{1}\left(t\right)\right)-K\left(t,z_{2}\left(t\right)\right)\left|\right|\\ & \leq M\lambda T||z_{1}\left(\omega \right)-z_{2}{\left(\omega \right)||}_{PC_{1-\eta }}+L_{p}\left|\right|z_{1}\left(\omega \right)-z_{2}\left(\omega \right){\left|\right|}_{PC_{1-\eta }}\\ & \leq \left(M\lambda T+L_{p}\right)\left|\right|z_{1}-z_{2}{\left|\right|}_{PC_{1-\eta }}\\ & \leq k_{1}\left|\right|z_{1}-z_{2}{\left|\right|}_{PC_{1-\eta }}. \end{array}$$ (3.7)

Also, for $z\in {ℵ}_{\gamma }$, $t\in (e_{k},t_{k+1}]$,

$$\begin{array}{cc} \left|\right|\left(G_{1}z_{1}\right)\left(t\right)-\left(G_{1}z_{2}\right)\left(t\right)\left|\right| & \leq (M(\frac{K_{I_{k}}t_{k+1}^{p_{1}}}{\Gamma (p_{1}+1)}+L_{p})+L_{p})\left|\right|z_{1}-z_{2}{\left|\right|}_{PC_{1-\eta }}\\ & \leq k_{1}\left|\right|z_{1}-z_{2}{\left|\right|}_{PC_{1-\eta }}. \end{array}$$ (3.8)

Also, for $t\in (t_{k},e_{k}]$, and $z\in {ℵ}_{\gamma }$,

$$\begin{array}{cc} & \left|\right|\left(G_{1}z_{1}\right)\left(t\right)-\left(G_{1}z_{2}\right)\left(t\right)\left|\right|\leq \frac{K_{I_{k}}t_{k+1}^{p_{1}}}{\Gamma (p_{1}+1)}\left|\right|z_{1}-z_{2}{\left|\right|}_{PC_{1-\eta }}\leq k_{1}\left|\right|z_{1}-z_{2}{\left|\right|}_{PC_{1-\eta }}. \end{array}$$ (3.9)

For any t∈I, $\left|\right|\left(G_{1}z_{1}\right)\left(t\right)-\left(G_{1}z_{2}\right)\left(t\right)\left|\right|\leq k_{1}\left|\right|z_{1}-z_{2}{\left|\right|}_{PC_{1-\eta }}$. Since $k_{1}<1,\;\;\;G_{1}$ is contracting operator.

**Step 3:** By step 1, it is clear that $G_{2}$ is bounded. To prove continuity, consider a sequence ${\left\{z^{n}\right\}}_{n=1}^{\infty }$ in ℵ_*γ*_ such that $z^{n}\to z$ in ℵ_*γ*_. For $t\in (0,t_{1}]$,

$$\begin{array}{c} \left|\right|\left(G_{2}z^{n}\right)\left(t\right)-\left(G_{2}z\right)\left(t\right)\left|\right|\\ \leq ||t^{\eta -1}\int_{0}^{t}{\left(t-\omega \right)}^{p_{1}-1}[F\left(\omega ,z^{n}\left(\omega \right)\right)-F\left(\omega ,z\left(\omega \right)\right)]d\omega ||\\ +||t^{\eta -1}\int_{0}^{t}{\left(t-\omega \right)}^{p_{1}-1}[Bu_{z^{n}}\left(\omega \right)-Bu_{z}\left(\omega \right)]d\omega ||\\ \leq M_{1}L_{f}\int_{0}^{t}\left|\right|z^{n}\left(\omega \right)-z\left(\omega \right)\left|\right|d\omega \\ +M_{1}M_{b}M_{w}^{1}\int_{0}^{t}[||S_{p_{1},p_{2}}\left(t\right)(\lambda \int_{0}^{T}z^{n}\left(\omega \right)d\omega -\lambda \int_{0}^{T}z\left(\omega \right)d\omega )||\\ +||K\left(t_{1},z^{n}\left(t_{1}\right)\right)-K\left(t,z\left(t\right)\right)\left|\right|+\int_{0}^{t_{1}}{\left(t-\omega \right)}^{p_{1}-1}||F\left(\omega ,z^{n}\left(\omega \right)\right)d\omega -F\left(\omega ,z\left(\omega \right)\right)\left|\right|d\omega ]d\tau \\ \leq M_{1}L_{f}\frac{t_{1}^{p_{1}}}{p_{1}}\left|\right|z^{n}-{z||}_{PC_{1-\eta }}+C_{1}[L_{p}+M_{1}L_{f}\frac{t_{1}^{p_{1}}}{p_{1}}]\left|\right|z^{n}-z{\left|\right|}_{PC_{1-\eta }}. \end{array}$$ (3.10)

Therefore, $\left|\right|\left(G_{2}z^{n}\right)\left(t\right)-\left(G_{2}z\right)\left(t\right)\left|\right|\to 0$ as *n* → ∞. Also, for $t\in (e_{k},t_{k+1}],\;\;k=1,2,\ldots ,N$,

$$\begin{array}{c} \left|\right|\left(G_{2}z^{n}\right)\left(t\right)-\left(G_{2}z\right)\left(t\right)\left|\right|\\ \leq ||t^{1-\eta }\int_{e_{k}}^{t}{\left(t-\omega \right)}^{p_{1}-1}[F\left(\omega ,z^{n}\left(\omega \right)\right)-F\left(\omega ,z\left(\omega \right)\right)]d\omega ||\\ +||t^{1-\eta }\int_{e_{k}}^{t}{\left(t-\omega \right)}^{p_{1}-1}[Bu_{z^{n}}\left(\omega \right)-Bu_{z}\left(\omega \right)]d\omega ||\\ \leq M_{1}L_{f}\int_{e_{k}}^{t}\left|\right|z^{n}\left(\omega \right)-z\left(\omega \right)\left|\right|d\omega \\ +M_{1}M_{b}M_{w}^{m}\int_{e_{k}}^{t}(||S_{p_{1},p_{2}}\left(t_{k+1}-e_{k}\right)[\frac{1}{\Gamma (p_{1})}\int_{t_{k}}^{e_{k}}{\left(e_{k}-\omega \right)}^{p_{1}-1}\\ \times [I_{k}\left(\omega ,z^{n}\left(t_{k}^{-}\right)\right)-I_{k}\left(\omega ,z\left(t_{k}^{-}\right)\right)]d\omega +[K\left(e_{k},z^{n}\left(e_{k}\right)\right)-K\left(e_{k},z\left(e_{k}\right)\right)]]||\\ +||K\left(t,z^{n}\left(t\right)\right)-K\left(t,z\left(t\right)\right)\left|\right|+\int_{e_{k}}^{t_{k+1}}{\left(t_{k+1}-\omega \right)}^{p_{1}-1}||F\left(\omega ,z^{n}\left(\omega \right)\right)d\omega -F\left(\omega ,z\left(\omega \right)\right)\left|\right|d\omega )d\tau \\ \leq M_{1}L_{f}\frac{t_{k+1}^{p_{1}}}{p_{1}}\left|\right|z^{n}-z{\left|\right|}_{PC_{1-\eta }}\\ +C_{2}(M(\frac{K_{I_{k}}t_{k+1}^{p_{1}}}{\Gamma (p_{1}+1)}+L_{p})+L_{p}+M_{1}L_{f}\frac{t_{k+1}^{p_{1}}}{p_{1}})\left|\right|z^{n}-z{\left|\right|}_{PC_{1-\eta }}. \end{array}$$ (3.11)

Hence, $\left|\right|\left(G_{2}z^{n}\right)\left(t\right)-\left(G_{2}z\right)\left(t\right)\left|\right|$ approaches to 0 as *n* approaches to ∞. Hence from (3.10) and (3.11) and for each t∈I, $\left|\right|\left(G_{2}z^{n}\right)\left(t\right)-\left(G_{2}z\right)\left(t\right)\left|\right|\to 0$ as *n* → ∞.

**Step 4:** $G_{2}$ is equicontinuous.

Take *τ*_1_ < *τ*_2_ on ℵ_*γ*_, and for $\tau_{1},\tau_{2}\in \left(0,t_{1}\right]$,

$$\begin{array}{c} \left|\right|\left(G_{2}z\right)\left(\tau_{2}\right)-\left(G_{2}z\right)\left(\tau_{1}\right)\left|\right|\\ \leq ||\tau_{2}^{\eta -1}\int_{0}^{\tau_{2}}{\left(\tau_{2}-\omega \right)}^{p_{1}-1}[F(\omega ,z(\omega ))+Bu(\omega )]d\omega \\ -\tau_{1}^{\eta -1}\int_{0}^{\tau_{1}}{\left(\tau_{1}-\omega \right)}^{p_{1}-1}[F(\omega ,z(\omega ))+Bu(\omega )]d\omega ||\\ \leq M_{1}L_{f}(\int_{0}^{\tau_{1}}[\tau_{2}^{\eta -1}{\left(\tau_{2}-\omega \right)}^{p_{1}-1}-\tau_{1}^{\eta -1}{\left(\tau_{1}-\omega \right)}^{p_{1}-1}]d\omega +\int_{\tau_{1}}^{\tau_{2}}\tau_{2}^{\eta -1}{\left(\tau_{2}-\omega \right)}^{p_{1}-1}d\omega )\\ +M_{1}M_{b}M_{w}^{1}(\left|\right|z_{t_{1}}\left|\right|+N)(\int_{0}^{\tau_{1}}[\tau_{2}^{\eta -1}{\left(\tau_{2}-\omega \right)}^{p_{1}-1}-\tau_{1}^{\eta -1}{\left(\tau_{1}-\omega \right)}^{p_{1}-1}]d\omega \\ +\int_{\tau_{1}}^{\tau_{2}}\tau_{2}^{\eta -1}{\left(\tau_{2}-\omega \right)}^{p_{1}-1}d\omega ). \end{array}$$ (3.12)

Similarly, For $\tau_{1},\tau_{2}\in \left(e_{k},t_{k+1}\right]$,

$$\begin{array}{c} \left|\right|\left(G_{2}z\right)\left(\tau_{2}\right)-\left(G_{2}z\right)\left(\tau_{1}\right)\left|\right|\\ \leq ||\tau_{2}^{\eta -1}\int_{e_{k}}^{\tau_{2}}{\left(\tau_{2}-\omega \right)}^{p_{1}-1}[F(\omega ,z(\omega ))+Bu(\omega )]d\omega \\ -\int_{e_{k}}^{\tau_{1}}{\left(\tau_{1}-\omega \right)}^{p_{1}-1}[F(\omega ,z(\omega ))+Bu(\omega )]d\omega ||\\ \leq M_{1}L_{f}(\int_{e_{k}}^{\tau_{1}}[\tau_{2}^{\eta -1}{\left(\tau_{2}-\omega \right)}^{p_{1}-1}-\tau_{1}^{\eta -1}{\left(\tau_{1}-\omega \right)}^{p_{1}-1}]d\omega +\int_{\tau_{1}}^{\tau_{2}}\tau_{2}^{\eta -1}{\left(\tau_{2}-\omega \right)}^{p_{1}-1}d\omega )\\ +M_{1}M_{b}M_{w}^{m}(\left|\right|z_{t_{k+1}}\left|\right|+N_{1})(\int_{e_{k}}^{\tau_{1}}[\tau_{2}^{\eta -1}{\left(\tau_{2}-\omega \right)}^{p_{1}-1}-\tau_{1}^{\eta -1}{\left(\tau_{1}-\omega \right)}^{p_{1}-1}]d\omega \\ +\int_{\tau_{1}}^{\tau_{2}}\tau_{2}^{\eta -1}{\left(\tau_{2}-\omega \right)}^{p_{1}-1}d\omega ). \end{array}$$ (3.13)

By (*H*3), $\left|\right|\left(G_{2}z\right)\left(\tau_{2}\right)-\left(G_{2}z\right)\left(\tau_{1}\right)\left|\right|\to 0$ as *τ*_2_ → *τ*_1_. Then $G_{2}$ is equicontinuous.

The countable subset $D_{0}={\left\{z_{n}\right\}}_{n=1}^{\infty }\subset D$, and by Lemma 2.4, we have

$$\begin{array}{cc} & \ell {\left(G_{2}\left(D\right)\right)}_{PC_{1-\eta }}\leq 2\;\ell {\left(G_{2}\left(D_{0}\right)\right)}_{PC_{1-\eta }}, \end{array}$$ (3.14)

where *D* is a bounded subset of ℵ_*γ*_. Since $G_{2}\left(D_{0}\right)\subset G_{2}\left({ℵ}_{\gamma }\right)$ is bounded and equicontinuous, by Lemma 2.6,

$$\begin{array}{cc} & \ell {\left(G_{2}\left(D_{0}\right)\right)}_{PC_{1-\eta }}\leq \underset{t\in (e_{k},t_{k+1}],\;\;k=0,1,2,\ldots ,N}{\text{max}}\ell {\left(G_{2}\left(D_{0}\right)\right)}_{PC_{1-\eta }}\left(t\right). \end{array}$$ (3.15)

Moreover, for $t\in (e_{k},t_{k+1}]$, (*H*4), (*H*7) and $G_{2}$, with Lemma 2.5, we have

$$\begin{array}{c} \begin{array}{cc} \ell \left(G_{2}\left(D_{0}\right)\right)\left(t\right) & \leq \ell (M_{1}\int_{e_{k}}^{t}[Bu(\omega ,{\left\{z_{n}\left(\omega \right)\right\}}_{n=1}^{\infty })+F(\omega ,{\left\{z_{n}\left(\omega \right)\right\}}_{n=1}^{\infty })]d\omega )\\ & \leq [2L_{u}^{*}\sigma^{*}M_{1}M_{b}M_{w}+2M_{1}L_{k}]\int_{e_{k}}^{t}\ell ({\left\{z_{n}\left(\omega \right)\right\}}_{n=1}^{\infty })d\omega \\ & \leq [2L_{u}^{*}\sigma^{*}M_{1}M_{b}M_{w}+2M_{1}L_{k}]\ell {\left(D\right)}_{PC_{1-\eta }}\left(t_{k+1}-e_{k}\right). \end{array} \end{array}$$ (3.16)

Then, by (3.14)–(3.16) and (*H*2),

$$\begin{array}{cc} \ell {\left(G_{2}\left(D\right)\left(t\right)\right)}_{PC_{1-\eta }} & \leq [2L_{u}^{*}\sigma^{*}M_{1}M_{b}M_{w}+2M_{1}L_{k}]\ell {\left(D\right)}_{PC_{1-\eta }}\left(t_{k+1}-e_{k}\right)\\ & \leq [4L_{u}^{*}\sigma^{*}M_{1}M_{b}M_{w}+4M_{1}L_{k}]\ell {\left(D\right)}_{PC_{1-\eta }}. \end{array}$$ (3.17)

Now, for any $t\in (e_{k},t_{k+1}]$, on *D* ∈ ℵ_*γ*_,

$$\begin{array}{cc} & \ell \left(G_{1}\left(D\right)\right)\leq [M\lambda +2L_{p}^{*}+M_{I}\left(M+1\right)]\ell \left(D\right). \end{array}$$ (3.18)

Also,

$$\begin{array}{c} \ell \left(G\left(D\right)\right)\leq \ell \left(G_{1}\left(D\right)\right)+\ell \left(G_{2}\left(D\right)\right)\\ \leq [M\lambda +2L_{p}^{*}+M_{I}\left(M+1\right)+4L_{u}^{*}\sigma^{*}M_{1}M_{b}M_{w}+4M_{1}L_{k}]\ell {\left(D\right)}_{PC_{1-\eta }}. \end{array}$$ (3.19)

Combining Lemma 3.1, and (3.3) and (3.19) it is clear that the mapping G from ℵ_*γ*_ to ℵ_*γ*_ is *κ*-set-contractive. Hence, the system G has a fixed point by Lemma 3.2. This completes the proof.

## 4 Optimal control

(*H*8) (i) The Lagrange function L:I×Y×U→R∪{∞} is Borel measurable;
    (ii) For t∈I, and for every $z_{1},z_{2}\in Y$, L(t,z,·) is convex on *U*;
    (iii) For almost all t∈I, L(t,·,·) is sequentially lower semi continuous on Y×U;
    (iv) For $c_{1}\geq 0,c_{2}>0$, $h\in L^{P}\left(I,R\right)$,
  $$L\left(t,z,u\right)\geq h\left(t\right)+c_{1}{\left||z|\right|}_{PC_{1-\eta }}+c_{2}{\left||u|\right|}^{P}.$$

This part deals with the verification of existence of optimal pair for the system (1.1) by sequencing technique as discussed in [46, 48]. Let the cost function(L) as:

$$I\left(z^{u},u\right)=\int_{0}^{T}L\left(t,z\left(t\right),u\left(t\right)\right)dt,\;\;\;\;\;\;u\in U_{ad}.$$

Define the admissible control function $U_{ad}$ as:

$$U_{ad}=\left\{u\in L^{P}\left(I,H\right);\;u\left(t\right)\in P\left(t\right),\;a.e.\;t\in I\right\},\;\;\;P>1,$$

where u(t) takes its values in S⊂U. A multivalued map $P:I\to PC_{1-\eta }$, is measurable as P(·)⊂S. It is clear that $U_{ad}$ is bounded, convex & closed with $U_{ad}=0$. Define the solution set

$$T\left(u\right)=\left\{z^{u}\in {ℵ}_{\gamma }:z^{u}\;\;u\in U_{ad}\right\}.$$

Also, the set of all $A_{ad}=\left\{\left(z^{u},u\right);\;u\in U_{ad};\;z^{u}\in T\left(u\right)\right\}$.

**Theorem 4.1**. *The system* (1.1) *is optimal controllable together with the assumptions (H1)-(H8) provided*

$$I\left({\tilde{z}}^{u^{0}},u^{0}\right)=\int_{0}^{T}L\left(t,{\tilde{z}}^{u^{0}}\left(t\right),u^{0}\left(t\right)\right)dt\leq I\left(z^{u},u\right),\;\;\forall \;\;\left(z^{u},u\right)\in A_{ad}.$$

*Proof*. Define $I\left(u\right)=\underset{z^{u}\in T\left(u\right)}{\text{inf}}I\left(z^{u},u\right)$. Initially we prove $I\left({\bar{z}}^{u},u\right)=I\left(u\right),\;\;\;z^{u}\in T\left(u\right)$. If I(u)=+∞ or T(u) has finite elements, the proof is trivial. Using (*H*8)(*iv*), I(u)>-∞. Let I(u)<∞. By infimum properties, a sequence ${\left\{z_{n}^{u}\right\}}_{n=1}^{\infty }\in T\left(u\right)$ satisfies $I\left(z_{n}^{u},u\right)\to I\left(u\right)$ as *n* → ∞. Using reflexive property, $\left\{u^{0}\right\}\in T\left(u\right)$ provided $u^{0}\in U_{ad}$.

For *n* ≥ 1,

$$\left(z_{n}^{u}\right)\left(t\right)=\{\begin{array}{c} S_{p_{1},p_{2}}\left(t\right)[\lambda \int_{0}^{T}z_{n}^{u}\left(\omega \right)d\omega +c-K\left(0,z\left(0\right)\right)]+K\left(t,z_{n}^{u}\left(t\right)\right)\\ +\int_{0}^{t}K_{p_{1}}\left(t-\omega \right)[Bu\left(\omega \right)+F(\omega ,z_{n}^{u}\left(\omega \right))]d\omega ,\;t\in \left(0,t_{1}\right];\\ S_{p_{1},p_{2}}\left(t-e_{k}\right)[\frac{1}{\Gamma (p_{1})}\int_{t_{k}}^{e_{k}}{\left(e_{k}-\omega \right)}^{p_{1}-1}I_{k}\left(\omega ,z_{n}^{u}\left(t_{k}^{-}\right)\right)d\omega -K\left(e_{k},z_{n}^{u}\left(e_{k}\right)\right)]\\ +K\left(t,z_{n}^{u}\left(t\right)\right)+\int_{e_{k}}^{t}K_{p_{1}}\left(t-\omega \right)[Bu\left(\omega \right)+F(\omega ,z_{n}^{u}\left(\omega \right))]d\omega ,\;t\in \left(e_{k},t_{k+1}\right], \end{array}$$

where

$$\left(z_{n}^{u}\right)\left(t\right)=\left(G_{1}z_{n}^{u}\right)\left(t\right)+\left(G_{2}z_{n}^{u}\right)\left(t\right)=\left(Gz_{n}^{u}\right)\left(t\right).$$

To prove $\left(Bz_{n}^{u}\right)\left(t\right):\left\{G_{2}\left(t\right);\;\;z_{n}^{u}\in ℵ\gamma \right\}$ is relatively compact in *PC*_1−*η*_ for each t∈I.

It is clear that $B\left(0\right):\{G_{2}\left(0\right);\;\;z_{n}^{u}\in ℵ\gamma \}$ is relatively compact. For any $u\in U,\;t\in I,\;\;\;z_{n}^{u}\in {ℵ}_{\gamma }$,

$$\left(G_{2}z_{n}^{u}\right)\left(t\right)=\int_{0}^{t}K_{p_{1}}\left(t-\omega \right)[Bu\left(\omega \right)+F(\omega ,z_{n}^{u}\left(\omega \right))]d\omega .$$

By (*H*3), and the property of admissible of control functions the set $W_{\epsilon }=\left\{K_{p_{1}}\left(t-\omega \right)[Bu\left(\omega \right)+F(\omega ,z_{n}^{u}\left(\omega \right))];0\leq e_{k}\leq t_{k}-\epsilon \right\}$ is relatively compact. Therefore, $\bar{W_{\epsilon }}$, the convex hull of $W_{\epsilon }$ is compact due to Lemma 2.3(ii). Using Lemma 2.5, we can conclude $\left(G_{2}^{\epsilon }z_{n}^{u}\right)\left(t\right)\in \bar{W_{\epsilon }}$ for all t∈I. Therefore $B_{\epsilon }\left(t\right):\left\{\left(G_{2}^{\epsilon }z_{n}^{u}\right)\left(t\right);\;\;z_{n}^{u}\in ℵ\gamma \right\}$ is relatively compact in *PC*_1−*η*_. For $t\in (0,t_{1}]$,

$$\begin{array}{c} \left|\right|\left(G_{2}z_{n}^{u}\right)\left(t\right)-\left(G_{2}^{\epsilon }z_{n}^{u}\right)\left(t\right)\left|\right|\\ \leq ||\int_{0}^{t}K_{p_{1}}\left(t-\omega \right)[F(\omega ,z_{n}^{u}\left(\omega \right))+Bu\left(\omega \right)]d\omega -\int_{0}^{t-\epsilon }[F(\omega ,z_{n}^{u}\left(\omega \right))+Bu\left(\omega \right)]d\omega ||\\ \leq \int_{t-\epsilon }^{t}K_{p_{1}}\left(t-\omega \right)[F(\omega ,z_{n}^{u}\left(\omega \right))+Bu\left(\omega \right)]d\omega \\ \leq M_{1}L_{f}\int_{t-\epsilon }^{t}\left|\right|z_{n}^{u}\left(\omega \right)||d\omega +M_{1}M_{b}{\left||u|\right|}_{L_{P}}. \end{array}$$

Similarly, for $t\in (e_{k},t_{k+1}]$,

$$\begin{array}{c} \left|\right|\left(G_{2}z_{n}^{u}\right)\left(t\right)-\left(G_{2}^{\epsilon }z_{n}^{u}\right)\left(t\right)\left|\right|\\ \leq ||\int_{e_{k}}^{t}K_{p_{1}}\left(t-\omega \right)[F(\omega ,z_{n}^{u}\left(\omega \right))+Bu\left(\omega \right)]d\omega -\int_{e_{k}}^{t-\epsilon }[F(\omega ,z_{n}^{u}\left(\omega \right))+Bu\left(\omega \right)]d\omega ||\\ \leq \int_{t-\epsilon }^{t}K_{p_{1}}\left(t-\omega \right)[F(\omega ,z_{n}^{u}\left(\omega \right))+Bu\left(\omega \right)]d\omega \\ \leq M_{1}L_{f}\int_{t-\epsilon }^{t}\left|\right|z_{n}^{u}\left(\omega \right)||d\omega +M_{1}M_{b}{\left||u|\right|}_{L_{P}}, \end{array}$$

implies that $\text{lim}\;\epsilon \to 0||\left(G_{2}z_{n}^{u}\right)\left(t\right)-\left(G_{2}^{\epsilon }z_{n}^{u}\right)\left(t\right)\left|\right|=0$. Hence $B_{\epsilon }\left(t\right)$, is a family of relatively compact sets. Moreover, $G_{1}z_{n}^{u}$ is bounded and equicontinuous in ℵ_*γ*_. By (3.16) and (3.18) we have

$$\begin{array}{cc} & \ell \left(z_{n}^{u}\right)\leq [M\lambda +2L_{p}^{*}+M_{I}\left(M+1\right)+4L_{u}^{*}\sigma^{*}M_{1}M_{b}M_{w}+4M_{1}L_{k}]\ell \left(z_{n}^{u}\right), \end{array}$$

leads to $\ell ({\left\{z_{n}^{u}\right\}}_{n=0}^{\infty })=0$ by using (3.3). Hence, ${\left\{z_{n}^{u}\right\}}_{n=1}^{\infty }$ is relatively compact in *PC*_1−*η*_. Assume ${\tilde{z}}^{u}$, a subsequence in *PC*_1−*η*_ of ${\left\{z_{n}^{u}\right\}}_{n=0}^{\infty }$ such that $z_{n}^{u}\to {\tilde{z}}^{u}$ as lim *n* → ∞. Moreover, by Lebesgue theorem and (*H*1), (*H*3), (*H*5)

$$\left({\tilde{z}}^{u}\right)\left(t\right)=\{\begin{array}{c} S_{p_{1},p_{2}}\left(t\right)[\lambda \int_{0}^{T}{\tilde{z}}^{u}\left(\omega \right)d\omega +c-K\left(0,z\left(0\right)\right)]+K\left(t,{\tilde{z}}^{u}\left(t\right)\right)\\ +\int_{0}^{t}K_{p_{1}}\left(t-\omega \right)[Bu\left(\omega \right)+F(\omega ,{\tilde{z}}^{u}\left(\omega \right))]d\omega ,\;t\in \left(0,t_{1}\right];\\ S_{p_{1},p_{2}}\left(t-e_{k}\right)[\frac{1}{\Gamma (p_{1})}\int_{t_{k}}^{e_{k}}{\left(e_{k}-\omega \right)}^{p_{1}-1}I_{k}\left(\omega ,{\tilde{z}}^{u}\left(t_{k}^{-}\right)\right)d\omega -K\left(e_{k},{\tilde{z}}^{u}\left(e_{k}\right)\right)]\\ +K\left(t,{\tilde{z}}^{u}\left(t\right)\right)+\int_{e_{k}}^{t}K_{p_{1}}\left(t-\omega \right)[Bu\left(\omega \right)+F(\omega ,{\tilde{z}}^{u}\left(\omega \right))]d\omega ,\;t\in \left(e_{k},t_{k+1}\right]. \end{array}$$

Then, ${\tilde{z}}^{u}\in T\left(u\right)$ is continuously embedded in $L^{1}\left(I,U\right)$, by Balder’s theorem [49] and (*H*8),

$$I\left(u\right)=\underset{n\to \infty }{\text{lim}}\int_{0}^{T}L\left(t,z_{n}^{u}\left(t\right),u\left(t\right)\right)dt\geq \int_{0}^{T}L\left(t,{\tilde{z}}^{u}\left(t\right),u\left(t\right)\right)dt=I\left({\tilde{z}}^{u},u\right)\geq I\left(u\right),$$

which shows $I\left({\tilde{z}}^{u},u\right)\to I\left(u\right)$. Therefore, I(u) reaches its least value at ${\tilde{z}}^{u}\in T\left(u\right)$ for every $u\in U_{ad}$.

Also, consider $u^{0}\in U_{ad}$ such that $I\left(u\right)=\underset{u\in U_{ad}}{\text{inf}}I\left(u\right)$. By the infimum property, ${\left\{u_{n}\right\}}_{n=0}^{\infty }\subseteq U_{ad}$ provided $\underset{n\to \infty }{\text{lim}}I\left(u_{n}\right)=\underset{u\in U_{ad}}{\text{inf}}I\left(u\right)$. Since ${\left\{u_{n}\right\}}_{n=0}^{\infty }$ in $L_{P}\left(I,U\right)$ is bounded for *P* > 1, $u^{0}\in L_{P}\left(I,U\right)$ and by relative compactness of $z_{n}^{u}$ there is a subsequence ${\tilde{z}}^{u^{0}}\in PC_{1-\eta }$ as $\underset{n\to \infty }{\text{lim}}z_{n}^{u}\to {\tilde{z}}^{u^{0}}$. Using Balder’s theorem [49] and the property that $PC_{1-\eta }\to L\left(I,U\right)$ is continuous, we conclude

$$\begin{array}{cc} \underset{u\in U_{ad}}{\text{inf}}I\left(u\right) & =\underset{n\to \infty }{\text{lim}}\int_{0}^{T}L\left(t,z_{n}^{u_{n}}\left(t\right),u_{n}\left(t\right)\right)dt\geq \int_{0}^{T}L\left(t,{\tilde{z}}^{u^{0}}\left(t\right),u^{0}\left(t\right)\right)dt=I\left({\tilde{z}}^{u^{0}},u^{0}\right)\\ & =I\left(u^{0}\right)\geq \underset{u\in U_{ad}}{\text{inf}}I\left(u\right). \end{array}$$

Therefore, $I\left(u\right)=\underset{u\in U_{ad}}{\text{inf}}I\left(u\right)$, leads that I attains its minimum at $u^{0}\in U_{ad}$. Subsequently, we have

$$I\left({\tilde{z}}^{u^{0}},u^{0}\right)=\underset{u\in U_{ad}}{\text{inf}}I\left(u\right)=\underset{\left(z^{u},u\right)\in A_{ad}}{\text{inf}}I\left(z^{u},u\right).$$

Hence, $\left({\tilde{z}}^{u^{0}},u^{0}\right)\in A_{ad}$. This completes the proof.

## 5 Application

Consider a nonlinear equation of the form given below to validate the outcome,

$$\begin{array}{c} D^{\frac{1}{2},\frac{2}{3}}[z\left(\varsigma ,\;t\right)-\text{exp}(z(\frac{3\varsigma t}{4}))]=\frac{\partial^{2}}{\partial t^{2}}[z\left(\varsigma ,\;t\right)-\text{exp}(z(\frac{3\varsigma t}{4}))]\\ +\int_{0}^{1}h\left(\varsigma ,t\right)u\left(\varsigma ,t\right)dt+\text{sin}[\text{exp}\left(\varsigma t\right)+z(\frac{2\varsigma t}{5})],\;\;\;\;\varsigma \in \left(0,3\right]\backslash \;\left(1,2\right],\\ z(\varsigma ,\;0)=z(\varsigma ,\;\pi )=0,\;\varsigma \in [1,2],\\ z\left(\varsigma ,\;t\right)=\frac{1}{\Gamma (\frac{1}{2})}\int_{t_{k}}^{t}\frac{1}{{\left(\varsigma -t\right)}^{\frac{1}{2}}}(\frac{z({\frac{1}{2}}^{-})}{20exp(t)+1})dt,\;\;\varsigma ,\;t\in \left[1,2\right],\\ I_{0}^{\frac{5}{6}}z\left(0,\;t\right)=\int_{0}^{3}z\left(\omega ,t\right)dt+5,\;\;\;t\in \left[0,\pi \right], \end{array}$$ (4.1)

with $Bu\left(\varsigma \right)\left(t\right)=\int_{0}^{1}h\left(\varsigma ,t\right)u\left(\varsigma ,t\right)dt$, & $p_{1}=\frac{1}{2},\;\;\;p_{2}=\frac{2}{3},\;\;\;\eta =\frac{5}{6}$. Assume $N=L^{2}\left[0,\;\pi \right]$ and A:D(A)⊂N→N by $Az=\frac{\partial^{2}}{\partial t^{2}}\left(z\right),\;$

$$\begin{array}{c} D\left(A\right)=\{z\in N,\;z_{t},z_{tt}\;\in N,\;z\left(\varsigma ,0\right)=z\left(\varsigma ,\pi \right)=0\}. \end{array}$$

It is clear that *A* is a strongly continuous semigroup and (S(ς)z) in N,

$$\left(S\left(\varsigma \right)z\right)\left(t\right)=\{\begin{array}{cc} \int_{0}^{\pi }M\left(\varsigma ,t-\omega \right)z\left(\omega \right)d\omega , & \varsigma >0,\\ z(t), & \varsigma =0, \end{array}$$

with

$$M\left(\varsigma ,t\right)=\sqrt{\frac{2}{\pi }}exp(-(\frac{t^{2}}{4\varsigma })),\;\;\;\varsigma >0,\;\;\;0<t<\pi ,$$

with z(ς)(t)=z(ς,t). This leads to the conclusion ||S(ς)||≤M. Let $U_{ad}={\left\{u\in U|||u|\right|}_{L^{2}\left[\left[0,3\right],\;U\right]}\leq 1\}.$ Hence

$$I\left(z,u\right)=\int_{t_{k}}^{t_{k+1}}\int_{0}^{\pi }{\left|z\left(\varsigma ,t\right)\right|}^{2}dtd\varsigma +\int_{t_{k}}^{t_{k+1}}\int_{0}^{\pi }{\left|u\left(\varsigma ,t\right)\right|}^{2}dtd\varsigma$$

related to the system (4.1) which correlates the system (1.1) with

$$I\left(z,u\right)=\int_{t_{k}}^{t_{k+1}}({\left||z\left(\varsigma \right)|\right|}^{2}+{\left||u\left(\varsigma \right)|\right|}_{U}^{2})d\varsigma .$$

Therefore, (*H*1)–(*H*8) satisfied. This completes the proof.

## 6 Conclusion

We examine the total controllability of non-instantaneous Hilfer fractional neutral system under integral boundary condition. By incorporating HFD with semigroup operator theory and Laplace transform technique, the integral solution is derived. Controllability outcomes were attained using Kuratowski’s measure with contraction theory. Furthermore, the sequencing technique has been used to discuss the existence of the optimal pair for the system. To confirm the derived consequences, an example is given. The concept can be extended to Hilfer stochastic differential equations.
